# Prevalence of Irritable Bowel Syndrome and Its Association With Anxiety Among Family Physicians in Primary Healthcare in Tabuk, Saudi Arabia

**DOI:** 10.7759/cureus.88138

**Published:** 2025-07-17

**Authors:** Wejdan Mohammed Alshehri, Hoda Mohamed Elhady, Asma Ali Alharbi, Wejdan A Alshehri, Arub M Albalawi, Nagham Mohammad Alkhrissi, Hadeel Ahmed Almutairi, Anwar Albalawi

**Affiliations:** 1 Department of Family Medicine, Tabuk Health Cluster, Tabuk, SAU; 2 Academic Affairs, Family Medicine Residency Training Program, Tabuk Health Cluster, Tabuk, SAU; 3 Department of Family Medicine, Ministry of Health, Saudi Arabia, Tabuk, SAU; 4 General Practice, King Fahad Specialist Hospital, Tabuk, SAU

**Keywords:** anxiety, family physicians, irritable bowel syndrome, primary healthcare, saudi arabia

## Abstract

Background: Irritable bowel syndrome (IBS) is a common functional gastrointestinal disorder influenced by multiple psychosocial factors, including anxiety. Physicians, particularly those working in primary healthcare settings, are exposed to high occupational stress, potentially increasing their risk for both anxiety and IBS.

Objective: This study aimed to determine the prevalence of IBS among family physicians in Tabuk, Saudi Arabia, and to explore its association with anxiety levels.

Methods: An analytical cross-sectional study was conducted between May 2024 and April 2025 among 300 family physicians working in primary healthcare centers (PHCCs) in Tabuk, Saudi Arabia. Participants were selected using a systematic random sampling technique. Data were collected using a self-administered online questionnaire that included the Rome IV criteria for IBS and the Generalized Anxiety Disorder-7 (GAD-7) scale. A pilot study was conducted with 20 physicians to validate the questionnaire. Data analysis was performed using IBM SPSS Statistics for Windows, version 28. Logistic regression was used to assess predictors of IBS. Statistical significance was set at p < 0.05.

Results: The prevalence of IBS among participants was 187 (62.3%), and 125 (41.7%) reported mild anxiety. Moderate anxiety was observed in 34 (11.3%) and severe anxiety in 28 (9.3%). Anxiety severity was significantly associated with IBS (p = 0.001). Physicians with mild anxiety had 5.5 times higher odds of IBS compared to those with minimal anxiety (odds ratio (OR): 5.5, 95% confidence interval (CI): 3.1-9.8). A positive family history of IBS (183, 61.0%) and lower BMI were also significantly associated with IBS prevalence. The job title showed a borderline association (p = 0.053).

Conclusion: This study revealed a high prevalence of IBS and anxiety among family physicians in Tabuk. Anxiety severity, family history of IBS, and BMI were significant predictors. These findings highlight the need for psychological screening and stress management programs for healthcare professionals.

## Introduction

Irritable bowel syndrome (IBS) stands out as a prevalent digestive disorder, impacting up to 20% of the general population and showing a higher occurrence in women [1,2]. While its precise origins remain unclear, current understanding highlights the significant involvement of subtle inflammation and immune system shifts in the development of IBS-related symptoms [3]. IBS is characterized by abdominal pain and changes in bowel habits [4]. Diagnosing IBS involves a thorough patient history of dietary patterns, medication use, past medical and surgical events, family history (including colorectal cancer or inflammatory bowel disease) [5], and psychosocial factors. Clinicians also look for "red flag" symptoms, such as gastrointestinal bleeding, unexplained anemia, or unintentional weight loss, alongside a focused physical examination, ultimately utilizing the Rome IV criteria for diagnosis [6,7].

Healthcare workers face immense psychological pressures due to the demanding nature of their profession, including long working hours, emotional strain, and frequent exposure to suffering and death [8,9]. These stressors increase vulnerability to psychological disorders such as anxiety, depression, burnout, post-traumatic stress disorder (PTSD), and substance use disorders.

Anxiety, one of the most prevalent mental health disorders, often coexists with IBS [10]. Studies suggest that individuals with IBS are significantly more likely to experience anxiety disorders compared to the general population [11-13]. This association reflects important implications for diagnosis, treatment, and patient outcomes. From a healthcare perspective, understanding the interplay between anxiety and IBS is crucial for developing effective, holistic approaches to patient care. Multidisciplinary treatment models that integrate psychological assessment and intervention alongside traditional gastrointestinal management are becoming increasingly essential [14].

However, there is a lack of research specifically addressing the prevalence of IBS and its association with anxiety among family physicians in Saudi Arabia, as most prior studies have focused on medical students or general populations. Therefore, this study aimed to determine the prevalence of IBS among family physicians in Tabuk, Saudi Arabia, and to evaluate its association with anxiety using validated diagnostic tools.

## Materials and methods

Study design and setting

An analytical cross-sectional study design was conducted in Tabuk Province, Kingdom of Saudi Arabia (KSA). The target population included family physicians working in primary healthcare centers (PHCCs) across Tabuk, encompassing general practitioners, residents, specialists, and consultants. The study was carried out over 12 months, from May 2024 to April 2025. Physicians practicing in other specialties or those diagnosed with inflammatory bowel disease or colon cancer were excluded from participation. These exclusions were applied to prevent diagnostic confounding with IBS symptoms. The total number of physicians in Tabuk is 513, comprising 233 family medicine physicians and 107 general practitioners. The required sample size was calculated to be 181 participants, based on a 95% confidence interval and a 5% margin of error, using an online sample size calculator.

Sampling technique

For sampling, Tabuk was divided into four health sectors: the King Fahd Health Sector, the King Khalid Health Sector, the King Salman Military Sector, and Tabuk University. From each sector, four primary healthcare centers were selected through simple random sampling. Within these centers, participants were selected using a systematic random sampling technique. Specifically, every third physician from an alphabetically ordered list was invited to participate.

Data collection procedure

Data were collected using a self-administered, pre-validated online questionnaire distributed via WhatsApp using Google Forms.

Study instrument

The questionnaire used in this study consisted of four main sections. The first section gathered sociodemographic data, including age, gender, nationality, marital status, job title, and body mass index (BMI). The second section assessed clinical characteristics, such as medical and family history, surgical history, and self-reported food sensitivities.

The third section employed selected items from the Rome IV Diagnostic Questionnaire (R4DQ), a standardized and validated tool used to diagnose functional gastrointestinal disorders (FGIDs), including IBS [6]. These selected sections were administered in English using the original validated version and were reprinted with permission from the Rome Foundation. The fourth section incorporated the Generalized Anxiety Disorder-7 (GAD-7) scale, a widely validated and reliable instrument for evaluating the severity of anxiety symptoms [7]. The GAD-7 demonstrated high internal consistency in this study, with a Cronbach’s alpha of 0.974 (95% CI: 0.969-0.978). The data collection tool primarily consisted of multiple-choice and closed-ended questions designed to ensure clarity and ease of response.

Pilot study

Before the main data collection, a pilot study was conducted on a sample of 20 family physicians working in PHCCs in Tabuk. The objective of the pilot study was to assess the clarity, relevance, and reliability of the questionnaire, as well as to evaluate the feasibility of the data collection process. Feedback from participants was used to identify and correct any ambiguities or difficulties in understanding the questions. The internal consistency of the GAD-7 scale used in the pilot was evaluated using Cronbach’s alpha, which showed excellent reliability with a value of 0.802 (95% CI: 0.756-0.863). Data obtained from the pilot study were not included in the final analysis but were instrumental in refining the questionnaire and improving the overall design of the main study.

Data analysis

Data were analyzed using the IBM SPSS Statistics for Windows, Version 28.0 (released 2021, IBM Corp., Armonk, NY). Descriptive statistics, including frequencies and proportions, were used to summarize the sociodemographic characteristics, clinical history, and responses to the GAD-7 questionnaire. The GAD-7 is a validated seven-item tool used to assess the severity of anxiety symptoms over the past two weeks. Each item is scored on a four-point Likert scale ranging from 0 (“not at all”) to 3 (“nearly every day”), producing a total score between 0 and 21. Anxiety severity was categorized into four levels, i.e., minimal, mild, moderate, and severe, based on standard cut-off values. The presence of IBS was determined using the Rome IV diagnostic criteria, which require recurrent abdominal pain associated with defecation, changes in stool frequency, and/or changes in stool form over the preceding three months, with symptom onset at least six months before diagnosis. Binary logistic regression was selected because the primary outcome variable (presence or absence of IBS) was binary. Bivariate analyses were conducted using Pearson’s chi-square test or Fisher’s exact test, where appropriate, to examine associations between IBS and independent variables such as demographic factors, anxiety levels, clinical history, and family history. A binary logistic regression model was then performed to identify independent predictors of IBS. Variables that showed a p-value <0.1 in bivariate analysis were included in the multivariate model. Adjusted odds ratios (AORs) with 95% confidence intervals (CIs) were calculated, and statistical significance was set at p <0.05. Multicollinearity among predictors was assessed using variance inflation factors, and no significant multicollinearity was detected.

## Results

Table 1 presents the sociodemographic characteristics of 300 family physicians working in PHCCs in Tabuk, Saudi Arabia. Most participants were female, 205 (68.3%), while males comprised a smaller proportion, 95 (31.7%). In terms of nationality, most physicians were Saudi nationals, i.e., 226 (75.3%), compared to non-Saudis (74; 24.7%). Regarding marital status, married physicians represented the largest group (186; 62.0%), followed by those who were single (104; 34.7%) and a small percentage who were divorced (10; 3.3%). Job titles varied, with residents making up the highest proportion (146; 48.7%), followed by specialists (57; 19.0%), consultants (50; 16.7%), and general practitioners (GPs) (47; 15.7%). Concerning body mass index (BMI), 188 (62.7%) of the physicians had a normal weight, 85 (28.3%) were overweight, and 27 (9.0%) were classified as obese.

**Table 1:** Sociodemographic characteristics of the family physicians in the primary healthcare centers (PHCCs) in Tabuk, Saudi Arabia (N = 300)

Sociodemographics	No	%
Gender		
Male	95	31.7%
Female	205	68.3%
Nationality		
Saudi	226	75.3%
Non-Saudi	74	24.7%
Marital status		
Single	104	34.7%
Married	186	62.0%
Divorced	10	3.3%
Job Title		
General practitioner (GP)	47	15.7%
Resident	146	48.7%
Specialist	57	19.0%
Consultant	50	16.7%
Body mass index		
Normal weight	188	62.7%
Overweight	85	28.3%
Obese	27	9.0%

Table 2 outlines family physicians’ personal and family medical history in the PHCCs in Tabuk. A minority of participants reported having chronic health problems (51; 17.0%), with the most common conditions being hypertension (18; 35.3%), anemia (13; 25.5%), and diabetes mellitus (9; 17.6%) among those with chronic illnesses. Less frequently reported conditions included chronic sinusitis, bronchial asthma, and polycystic ovary syndrome, each affecting fewer than 6.0% of those with chronic illnesses. Regarding food sensitivity, only 32 (10.7%) physicians reported having some form of sensitivity, with wheat (12; 37.5%) and lactose intolerance (9, 28.1%) being the most commonly identified triggers. Surgical history was reported by 76 (25.3%) physicians. The most frequently performed procedures were cesarean section (32; 42.1%), tonsillectomy (14; 18.4%), and appendicectomy (11; 14.5%). Notably, a significant proportion of participants, 183 (61.0%), reported having a family history of IBS.

**Table 2:** Personal and family medical history of family physicians in the primary healthcare centers (PHCCs) in Tabuk, Saudi Arabia (N = 300)

Data	No	%
Do you have any chronic health problems?		
Yes	51	17.0%
No	249	83.0%
Diseases		
Hypertension	18	35.3%
Anemia	13	25.5%
Diabetes mellitus	9	17.6%
Chronic sinusitis	3	5.9%
Hypertension, Anemia	3	5.9%
Bronchial asthma	2	3.9%
Polycystic ovary syndrome	2	3.9%
Epilepsy	1	2.0%
Do you have food sensitivity?		
Yes	32	10.7%
No	268	89.3%
What type of food triggers your sensitivity?		
Wheat	12	37.5%
Lactose intolerance	9	28.1%
Lactose	3	9.4%
Spicy	3	9.4%
guava	2	6.3%
Shrimps	2	6.3%
Fatty acids	1	3.1%
Have you had any surgery before?		
Yes	76	25.3%
No	224	74.7%
Type of surgery		
Caesarian section	32	42.1%
Tonsillectomy	14	18.4%
Appendicectomy	11	14.5%
Cholecystectomy	8	10.5%
Gastric bypass	3	3.9%
Brain surgery	2	2.6%
FESS	2	2.6%
Hernioplasty	2	2.6%
Inguinal hernia	2	2.6%
Do you have a family history of IBS?		
Yes	183	61.0%
No	117	39.0%

**Figure 1:**
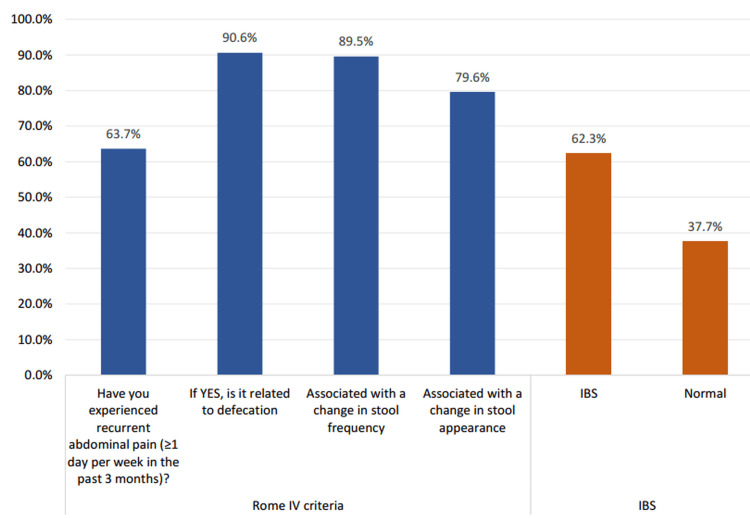
Prevalence and diagnostic criteria for irritable bowel syndrome (IBS) among family physicians in primary healthcare centers (PHCCs) in Tabuk, Saudi Arabia (N = 300)

Figure 1 shows the distribution of recurrent abdominal pain and the application of the Rome IV criteria among the study participants. A total of 191 (63.7%) participants reported experiencing recurrent abdominal pain (≥1 day per week in the past three months). Among these, 173 (90.6%) indicated that the pain was related to defecation, 171 (89.5%) reported an association with changes in stool frequency, and 152 (79.6%) noted changes in stool appearance. Based on the fulfillment of Rome IV criteria, 187 (62.3%) participants were classified as having IBS, while 113 (37.7%) were categorized as normal.

**Figure 2:**
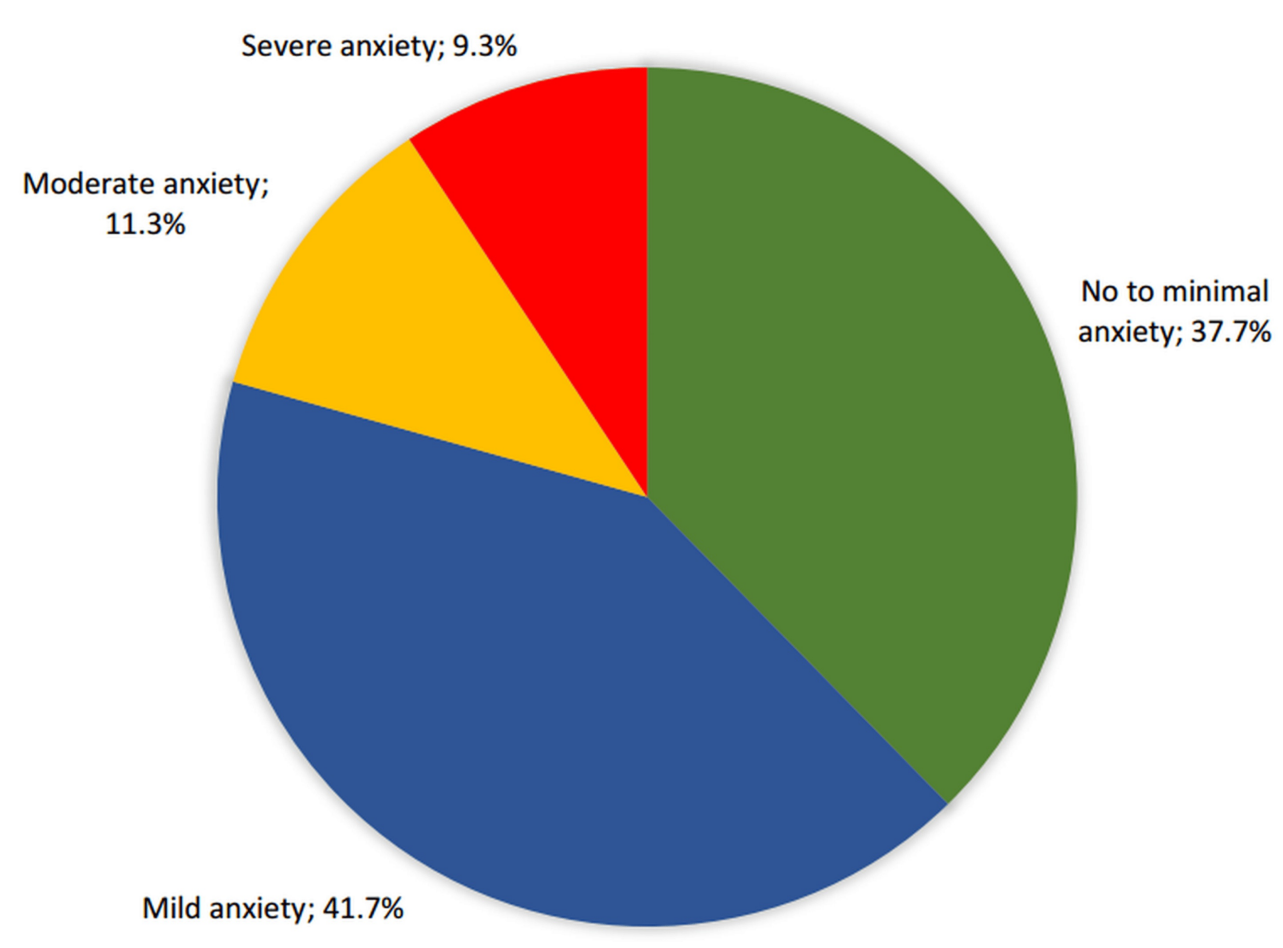
Severity levels of anxiety among family physicians based on GAD-7 scores (N = 300)

Figure 2 illustrates the proportion of anxiety severity categories among the physicians. The majority of participants reported some level of anxiety, with 125 (41.7%) experiencing mild anxiety and 62 (20.6%) experiencing moderate to severe anxiety, specifically, 34 (11.3%) with moderate and 28 (9.3%) with severe anxiety. Only 113 (37.7%) participants fell into the no to minimal anxiety category.

Table 3 presents the frequency of anxiety symptoms among family physicians using the GAD-7 scale. The most commonly reported symptom was feeling nervous, anxious, or on edge, experienced for at least several days by 224 (74.3%) participants, with 20 (6.7%) experiencing it nearly every day. Similarly, 196 (65.3%) reported being unable to stop or control worrying for several days or more, and 76 (25.3%) experienced it more than half the days or nearly every day. A total of 205 (68.3%) reported worrying too much about different things on several days or more, with 27 (9.0%) experiencing this symptom nearly every day. Trouble relaxing was common, with 61 (20.4%) experiencing it more than half the days or nearly every day. Symptoms of restlessness were reported by 54 (18.0%) participants for more than half the days or nearly daily. Irritability was reported by 196 (65.4%) participants on several days or more, including 24 (8.0%) nearly every day. Finally, 175 (58.3%) reported feeling afraid as if something awful might happen for several days or more, including 14 (4.7%) nearly every day.

**Table 3:** Frequency of anxiety symptoms among family physicians based on GAD-7 items (N = 300)

Items	Not at all		Several days		Over half days		Nearly every day	
	No	%	No	%	No	%	No	%
Feeling nervous, anxious, or on edge	77	25.7%	151	50.3%	52	17.3%	20	6.7%
Not being able to stop or control worrying	104	34.7%	130	43.3%	48	16.0%	18	6.0%
Worrying too much about different things	95	31.7%	131	43.7%	47	15.7%	27	9.0%
Trouble relaxing	95	31.7%	144	48.0%	38	12.7%	23	7.7%
Being so restless that it’s hard to sit still	124	41.3%	122	40.7%	42	14.0%	12	4.0%
Becoming easily annoyed or irritable	104	34.7%	134	44.7%	38	12.7%	24	8.0%
Feeling afraid as if something awful might happen	125	41.7%	127	42.3%	34	11.3%	14	4.7%

Table 4 examines the association between various sociodemographic and clinical factors and the presence of IBS. Marital status was significantly related to IBS (p = 0.049), with a higher prevalence among married individuals (121; 65.1%), compared to single (63; 60.6%) and divorced (3; 30.0%) participants. Job title also demonstrated a strong association (p = 0.001), with residents (104; 71.2%) and specialists (39; 68.4%) showing the highest prevalence of IBS, while general practitioners reported the lowest (14; 29.8%). The strongest association was found with a family history of IBS (p = 0.001), as 136 (74.3%) of those with a positive family history were diagnosed with IBS, compared to 51 (43.6%) among those without such a history.

**Table 4:** Association between sociodemographic and clinical factors and irritable bowel syndrome (IBS) among family physicians in primary healthcare centers (PHCCs), Tabuk, Saudi Arabia (N = 300) P: Pearson X2 test. ^: Exact probability test. * P < 0.05 (significant)

Factors	IBS				p-value
	IBS		Normal		
	No	%	No	%	
Gender					.280
Male	55	57.9%	40	42.1%	
Female	132	64.4%	73	35.6%	
Nationality					.755
Saudi	142	62.8%	84	37.2%	
Non-Saudi	45	60.8%	29	39.2%	
Marital status					.049*^
Single	63	60.6%	41	39.4%	
Married	121	65.1%	65	34.9%	
Divorced	3	30.0%	7	70.0%	
Job Title					.001*
General Practitioner (GP)	14	29.8%	33	70.2%	
Resident	104	71.2%	42	28.8%	
Specialist	39	68.4%	18	31.6%	
Consultant	30	60.0%	20	40.0%	
Body mass index					.080
Normal weight	126	67.0%	62	33.0%	
Overweight	45	52.9%	40	47.1%	
Obese	16	59.3%	11	40.7%	
Do you have any chronic health problems?					.376
Yes	29	56.9%	22	43.1%	
No	158	63.5%	91	36.5%	
Do you have food sensitivity?					.118^
Yes	24	75.0%	8	25.0%	
No	163	60.8%	105	39.2%	
Have you had any surgery before					.320
Yes	51	67.1%	25	32.9%	
No	136	60.7%	88	39.3%	
Do you have a family history of IBS?					.001*
Yes	136	74.3%	47	25.7%	
No	51	43.6%	66	56.4%	

Table 5 demonstrates a statistically significant association between anxiety severity and the presence of IBS (p = 0.001). Compared to participants with minimal anxiety, those with mild anxiety had significantly higher odds of IBS (OR = 5.5, 95% CI: 3.1-9.8). Individuals with moderate anxiety also showed increased odds (OR = 3.0, 95% CI: 1.4-6.9), and those with severe anxiety had similarly elevated odds (OR = 3.1, 95% CI: 1.3-7.4).

**Table 5:** Association between anxiety severity and irritable bowel syndrome (IBS) among family physicians in primary healthcare centers (PHCCs), Tabuk, Saudi Arabia (N=300) P: Pearson X2 test, OR: odds ratio, CI: confidence interval, * P < 0.05 (significant)

Anxiety level	IBS				p-value	OR (95% CI)
	IBS		Normal			
	No	%	No	%		
No to minimal anxiety	46	40.7%	67	59.3%	.001*	1
Mild anxiety	99	79.2%	26	20.8%		5.5 (3.1-9.8) *
Moderate anxiety	23	67.6%	11	32.4%		3.0 (1.4-6.9) *
Severe anxiety	19	67.9%	9	32.1%		3.1 (1.3-7.4) *

Table 6 presents the results of multiple logistic regression analysis identifying factors associated with the presence of IBS among family physicians. Anxiety severity remained a strong independent predictor (AOR = 1.82, 95% CI: 1.34-2.47). A positive family history of IBS was also significantly associated (adjusted OR = 0.28, 95% CI: 0.17-0.47), indicating lower odds among those without such a history. BMI showed a significant inverse association (AOR = 0.64, 95% CI: 0.42-0.98). Job title approached significance (AOR = 1.32, 95% CI: 1.00-1.76; p = 0.053). Neither food sensitivity (p = 0.495) nor marital status (p = 0.513) showed a statistically significant association with IBS.

**Table 6:** Multiple logistic regression model for predictors of irritable bowel syndrome (IBS) among the study participants (N = 300) OR_a_: adjusted odds ratio, CI: confidence interval. * P < 0.05 (significant)

Factors	p-value	OR_A_	95% CI	
			Lower	Upper
Anxiety severity	.001*	1.82	1.34	2.47
MS	.513	0.84	0.49	1.43
Job	.053	1.32	1.00	1.76
FH	.001*	0.28	0.17	0.47
BMI	.039*	0.64	0.42	0.98
Food sensitivity	.495	0.73	0.30	1.79

## Discussion

The present study aimed to assess the prevalence of IBS and its association with anxiety among family physicians working in PHCCs in Tabuk, Saudi Arabia. The majority of participants were female, Saudi nationals, and married, with residents constituting the largest professional group. Most physicians had a normal BMI, although a considerable proportion were overweight or obese. A minority reported chronic health conditions, primarily hypertension, anemia, and diabetes mellitus. Food sensitivities were uncommon, with wheat and lactose intolerance identified as the most frequent triggers. Surgical history was reported by approximately one-quarter of participants, with cesarean section being the most frequently performed procedure. In addition, a significant proportion of physicians reported a family history of IBS, suggesting potential hereditary or shared environmental influences.

A considerable proportion of participants met the Rome IV diagnostic criteria for IBS, with many reporting recurrent abdominal pain related to bowel habits. The observed prevalence appears higher than that reported in some studies conducted among the general Saudi population, such as the study by AlAmeel et al., which found a prevalence of 16.3% [15]. However, the results align more closely with studies focusing on healthcare professionals, likely reflecting the unique occupational stressors inherent in medical practice. For instance, research among medical students and interns in Riyadh reported elevated IBS rates attributed to academic and clinical stress [16], while a study in Jeddah demonstrated higher prevalence among physicians and nurses compared to non-medical professionals [17].

International evidence corroborates these findings, indicating that IBS is prevalent among individuals exposed to chronic stress and irregular work schedules. Meta-analyses have confirmed that high-stress occupations, such as healthcare, are associated with a greater risk of developing functional gastrointestinal disorders [18]. Studies conducted in Europe and North America have also linked IBS to occupational burnout, sleep disturbances, and irregular eating habits, factors frequently encountered among physicians [19]. For example, research in Canada reported an IBS prevalence of 22% among medical students [20], while studies among nurses have documented prevalence rates exceeding 30%, with even higher rates observed among those working rotating shifts [21].

Regarding anxiety, a substantial proportion of family physicians in this study reported symptoms ranging from mild to severe, with more than 60% experiencing at least some degree of anxiety. This finding is consistent with previous research demonstrating the heightened vulnerability of healthcare professionals to anxiety due to factors such as demanding workloads, emotional strain, and irregular schedules, both within Saudi Arabia [22-25] and internationally [26,27].

The analysis revealed a statistically significant association between anxiety severity and the presence of IBS. Notably, even mild anxiety was associated with a more than fivefold increase in the likelihood of having IBS compared to minimal anxiety. This association persisted across moderate and severe anxiety categories, albeit with slightly attenuated odds ratios, suggesting a potential threshold effect rather than a linear dose-response relationship. This observation aligns partially with prior studies, such as the meta-analysis by Fond et al., which also demonstrated a significant association between anxiety and IBS risk. However, the pronounced effect of mild anxiety observed in this study may reflect the unique stressors faced by physicians or the possibility that mild anxiety symptoms are more readily acknowledged than severe distress. Alternatively, this pattern could be influenced by coping mechanisms among physicians with more severe anxiety or by sample distribution effects and reporting biases. The plateau in risk at higher anxiety levels warrants further investigation to clarify these possibilities.

In addition, unmeasured confounding factors such as shift work, burnout, and sleep disorders may have contributed to the observed association between anxiety and IBS in this population. Future research should aim to account for these variables to improve the understanding of these complex relationships. From a clinical perspective, these findings highlight the importance of incorporating mental health screening and support services into occupational health programs for physicians to facilitate early identification and management of anxiety-related gastrointestinal symptoms.

Limitations

This study has several limitations that should be considered when interpreting the findings. The cross-sectional design precludes any causal inferences; more precisely, temporal or directional relationships between anxiety and IBS cannot be established. The use of self-administered questionnaires may have introduced recall bias, which is likely more relevant in this sample due to the reliance on recollection of symptoms, and social desirability bias, potentially affecting the accuracy of responses. Additionally, conducting the survey online may have limited participation to physicians comfortable with digital platforms, thereby introducing selection bias. The non-response rate could not be precisely determined, which represents a limitation that may have affected generalizability. The study was carried out in a single province, which may limit the generalizability of the results to other regions in Saudi Arabia. Finally, although validated tools such as the Rome IV criteria and GAD-7 scale were utilized, the reliance on self-reported measures may have underestimated or overestimated the true prevalence of anxiety and IBS.

## Conclusions

This study revealed a high prevalence of IBS and significant levels of anxiety, with about two-thirds experiencing at least mild anxiety. Key findings indicate a strong, statistically significant association between increasing anxiety severity and a higher likelihood of IBS. Specifically, physicians with mild anxiety were 5.5 times more likely to have IBS compared to those with minimal anxiety. Furthermore, a family history of IBS was strongly linked to a higher prevalence of the condition. Based on these findings, it is recommended that healthcare providers in primary care settings recognize the significant interplay between psychological well-being, particularly anxiety, and the prevalence of IBS among physicians. Further research could explore specific stress factors within the medical profession that might contribute to this relationship. Support systems and mental health services may be worth exploring in future interventional studies to better address these issues. Structured workplace interventions, such as stress management workshops, peer support groups, and confidential mental health services tailored for physicians, may also be valuable components of occupational health strategies in primary care settings. It should be noted that the cross-sectional design precludes causal inferences, and the observed association does not imply a causal relationship.

## Figures and Tables

**Figure 3:**
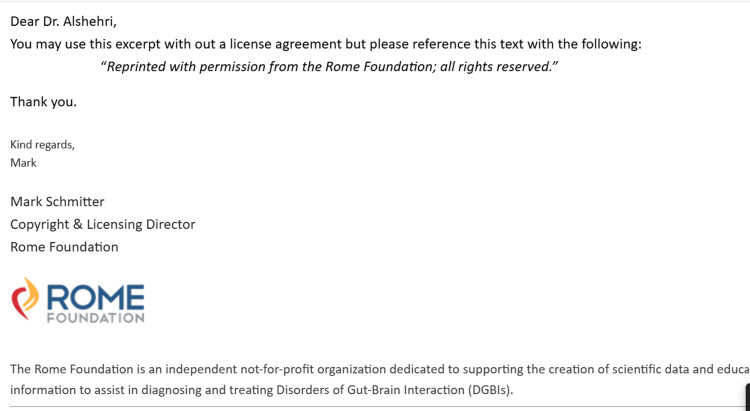
Permission Confirmation from Rome Foundation
